# [Retracted] Emodin promotes the arrest of human lymphoma Raji cell proliferation through the UHRF1-DNMT3A-∆Np73 pathways

**DOI:** 10.3892/mmr.2026.13968

**Published:** 2026-07-20

**Authors:** Yun Lin, Weiming Chen, Zhihong Wang, Pengwei Cai

Mol Med Rep 16: 6544–6551, 2017; DOI: 10.3892/mmr.2017.7423

Following the publication of the above paper, it was drawn to the Editor's attention by a concerned reader that, in comparing the western blot data shown in Figs. 2B and 4B, a strong similarity was noted comparing the Active-caspase 3 band in the ‘6.25’ lane of Fig. 2B with the NIRF band in the ‘6.25’ lane of Fig. 4B; moreover, the ICBP87 bands in the ‘Control’ and ‘6.25’ lanes of Fig. 6B also appeared to be strikingly similar. Finally, a pair of the DNMT1 bands shown for the western blots in Fig. 6B were very similar to a pair of the DNMT3B blots in the same figure part.

Following an independent investigation of this paper in the Editorial Office, the concerns raised by the reader were judged to be well-founded; therefore, the Editor of *Molecular Medicine Reports* has decided that this paper should be retracted from the Journal on account of a lack of confidence in the presented data. The authors were asked for an explanation to account for these concerns, but the Editorial Office did not receive a reply. The Editor apologizes to the readership for any inconvenience caused.

